# Recent Developments in Diabetic Retinal Neurodegeneration: A Literature Review

**DOI:** 10.1155/2020/5728674

**Published:** 2020-12-07

**Authors:** Shani Pillar, Elad Moisseiev, Jelizaveta Sokolovska, Andrzej Grzybowski

**Affiliations:** ^1^Department of Ophthalmology, Meir Medical Center, Kfar Saba, Israel; ^2^Sackler School of Medicine, Tel Aviv University, Tel Aviv, Israel; ^3^Faculty of Medicine, University of Latvia, Riga, Latvia; ^4^Department of Ophthalmology, University of Warmia and Mazury, Olsztyn, Poland; ^5^Institute for Research in Ophthalmology, Foundation for Ophthalmology Development, Poznan, Poland

## Abstract

Neurodegeneration plays a significant role in the complex pathology of diabetic retinopathy. Evidence suggests the onset of neurodegeneration occurs early on in the disease, and so a greater understanding of the process is essential for prompt detection and targeted therapies. Neurodegeneration is a common pathway of assorted processes, including activation of inflammatory pathways, reduction of neuroprotective factors, DNA damage, and apoptosis. Oxidative stress and formation of advanced glycation end products amplify these processes and are elevated in the setting of hyperglycemia, hyperlipidemia, and glucose variability. These key pathophysiologic mechanisms are discussed, as well as diagnostic modalities and novel therapeutic avenues, with an emphasis on recent discoveries. The aim of this article is to highlight the crucial role of neurodegeneration in diabetic retinopathy and to review the molecular basis for this neuronal dysfunction, its diagnostic features, and the progress currently made in relevant therapeutic interventions.

## 1. Introduction

Diabetic retinopathy is a major cause of preventable vision impairment and blindness worldwide, with increasing prevalence during recent decades [1, 2]. Traditionally, vasculopathy has been considered the primary pathophysiologic mechanism responsible for diabetic retinopathy (DR). However, in recent years, the role of diabetic retinal neurodegeneration (DRN) is increasingly evident and quite possibly supersedes that of vasculopathy as the primary pathogenic event of the disease. Indeed, it has been suggested that DRN is not only a possible biomarker for early development of the vasculopathy that constitutes DR but rather that DRN is in fact a causal factor in the development of DR [3–7]. The term diabetic retinal disease (DRD) is used to integrate the retinal microvasculopathy and retinal neuropathy caused by diabetes [8]. As current focus of medical practice, in terms of early detection and treatment of DRD, lies on the vascular component of DR, new discoveries regarding DRN's significance may lead to a paradigm shift. In this review, we aim to provide a comprehensive and up-to-date overview of the rapidly expanding body of work elucidating DRN's role in DRD and its effect on diagnostics and treatment.

## 2. Methods

The PubMed and Medline databases were the main resources used to conduct the medical literature search. An extensive search was conducted to identify relevant articles concerning DRN published up to March 31, 2020. Emphasis was placed on recent articles, published since January 1, 2018, but earlier articles were also included if they provided significant information to the understanding of DRN. The following keywords were used in various combinations: diabetic retinal neurodegeneration, neurodegenerative, neurodegeneration, neuroprotective, diabetes, diabetic retinopathy, diabetic retinal disease, diabetic macular edema, and diabetic eye disease. We included original studies and reviews that described incidence, pathogenesis, imaging, and therapies of retinal neurodegeneration in diabetes. Case reports were excluded. Of the studies retrieved by this method, we reviewed all publications in English and those having English abstracts. Other articles cited in the reference lists of identified publications were considered as a potential source of information. No attempts were made to discover unpublished data.

## 3. DRN Pathophysiology

### 3.1. DRN Basic Pathophysiology

Dysfunction of the retinal “neurovascular unit” (NVU) is key in the development of DRN. The term NVU refers to the intricate physical and functional relationship between neurons, glia, and vasculature in the central nervous system. In the retina, it forms the blood-retinal barrier (BRB) and maintains energy homeostasis and neurotransmitter regulation [9, 10]. The retinal NVU is damaged early in the progression of diabetes, as a result of processes of innate immunity, the complement system, and microglia activated by the disease [11]. Such damage is expressed by reduced functional reactivity, which may be detected prior to clinical appearance of DR changes [12–14]. Subsequent impairment in the NVUs leads to breakdown of the BRB and vascular leakage, with manifest retinopathy [9, 15]. The breakdown of the BRB is the culmination of processes governed by the secretion of many factors, among which are vascular endothelial growth factor (VEGF), proinflammatory cytokines (e.g., IL-1*β*, TNF-*α*, IL-6, and monocyte chemoattractant protein-1 (MCP-1)), and components of complement. These are variously secreted from RPE, glia, and immune cells [15]. In the late stages of DR, immune privilege is compromised, and the retina is infiltrated by circulating immune cells and serum proteins, further damaging blood vessels and neurons. Furthermore, even after the BRB is repaired, the blood-derived immune stimulators and responders may remain in the neuronal retina [16].

The impairment of the neurosensory retina in diabetes is governed by various mechanisms, which may be classified as inflammatory, metabolic, and genetic/epigenetic. Principal components include imbalance of neurotrophic factors, oxidative stress, and glial reactivity [17, 18]. The latter pertains to the activation and proliferation of astrocytes, Müller cells, and microglia in the diabetic retina, causing secretion of proinflammatory mediators and neurotoxic factors, with subsequent reactive gliosis, diminished retinal neuronal function, and neural-cell apoptosis [19–22]. Early-on in diabetes, changes in astrocytes are observed, such as a decrease in cell number and altered protein expression profile, coincide with inner retinal hypoxia and functional deficits in ganglion cell responses [23]. Müller cells' dysfunction due to chronic hyperglycemia causes them to release a large variety of growth factors and cytokines. This affects vascular dysfunction and angiogenesis but also serves to protect glia cells and retinal neurons [24]. Reactive Müller cells are thought to be initially neuroprotective but consequently may contribute to neuronal degeneration. This is owing to various dysfunctional Müller cell faculties, such as malfunction of glutamate uptake, and expression of nucleoside triphosphate diphosphohydrolase 1 (NTPDase1), enabling extracellular adenosine formation [25]. Microglia, the retinal macrophages, are activated in diabetes due to a complex interplay between hyperglycemia, oxidative stress, leukostasis, and vascular leakage. In turn, microglia increase proliferation and migration and demonstrate transcriptional changes, causing release of various proinflammatory mediators, including cytokines, chemokines, caspases, and glutamate. This results in apoptosis of retinal neurons, consequential thinning of the nerve fiber layer, and eventual visual loss [26–29]. Multifocal electroretinogram (mfERG) is most commonly used in studies to unveil the functional ramifications of DRN, even in patients with no DR or mild nonproliferative DR (NPDR) [30–33].

### 3.2. Recent Findings in DRN Pathophysiology

Galectin-3 regulates several biological processes, including ones involved in inflammation, oxidative stress, and apoptosis. It has been linked to diabetes' development and identified as a biomarker for prediabetes and diabetes [34, 35]. In streptozotocin- (STZ-) induced diabetic mice, galectin-3 knockout correlated with less macrophage infiltration/proliferation and less activation of astrocytes and microglia in the optic nerve, as well as less retinal ganglion cell (RGC) death and a higher number of myelinated nerve fibers [36]. These findings indicate galectin-3's involvement in stimulation of neuroinflammation and neurodegeneration in the diabetic retina [18].

Serine racemase (SRR) and its product, D-serine, are known to contribute to neurotoxicity, through serine's activity as an endogenous coagonist of the N-methyl-D-aspartate receptor (NMDA-R), a mediator of glutamate excitotoxicity. Previous studies show that increased retinal levels of SRR and D-serine are correlated with DRD [37, 38]. Recently, this link has been further substantiated owing to studies demonstrating an attenuation of retinal neurodegeneration in diabetic mice with SRR deletion or loss-of-function mutation [39, 40].

The stress response protein regulated in development and DNA damage-response 1 (REDD1), known to promote neuronal apoptosis, was previously demonstrated to be overexpressed in response to hyperglycemia in the retina of diabetic rodents [41]. A recent study elucidated the protein's importance in neurodegeneration. It was found that cell death occurred concomitantly with REDD1 overexpression in hyperglycemic conditions in retinal cell cultures, whereas REDD1-deficient cells were not driven to cell death by hyperglycemia. Similar results were exhibited in diabetic mice models, where retinal cell apoptosis, as well as functional deficiencies in visual acuity and contrast sensitivity, were avoided in REDD1-deficient diabetic mice [42].

The microtubule-associated protein tau is a critical mediator of neurotoxicity in neurodegenerative diseases, such as Alzheimer's disease (AD), but has not been previously studied in association with DRN. In a study of high-fat diet- (HFD-) induced diabetes mice models, hyperphosphorylated tau was found to cause vision deficits and synapse loss of RGCs and eventually retinal microvasculopathy and RGCs apoptosis [43].

Several neuroprotective factors were recently established to be associated with DRN: diabetic mice models were found to have reduced levels of *α*A-crystallin (molecular chaperone, regulating neuronal cell survival in multiple neurodegenerative conditions) [44], SIRT6 (a NAD-dependent sirtuin deacylase, known to modulates aging, energy metabolism, and neurodegeneration) [45], thioredoxin (antioxidant involved in antiapoptosis and transcriptional regulation) [46, 47], and ciliary neurotrophic factor (CNTF) [48]. Of note, CNTF is known to enhance survival of photoreceptors and RGCs and has broad neuroprotective effects on damaged retinas. Another known neuroprotectant, X-box binding protein 1 (XBP1), was recently studied using a conditional retina-specific knockout mouse line. The study demonstrated that depletion of XBP1 in retinal neurons results in early onset retinal function decline, loss of RGCs and photoreceptors, disrupted photoreceptor ribbon synapses, and Müller cell activation after induction of diabetes [49]. Interestingly, both XBP1 and *α*A-crystallin are involved in the regulation of the unfolded protein response or endoplasmic reticulum stress response, in which they play key roles to prevent protein aggregation and subsequent cell toxicity and cell death. Recent findings regarding the dysregulation of the L-arginine pathway in plasma samples from type 2 diabetic patients with PDR [50, 51] may lead to novel therapeutic avenues using substances such as arginase 1 for treatment of DRN and other ischemic retinopathies [52].

One factor which stands out in its increasingly recognized importance to neurodegenerative processes is Sigma1 receptor (Sig1R) [53–58]. It is a pluripotent modulator with a number of biological functions, many of which are relevant to retinal disease, including involvement in calcium regulation, modulation of oxidative stress, ion channel regulation, and molecular chaperone activity. Several compelling studies have provided evidence of powerful in vivo neuroprotective effects against ganglion cell loss as well as photoreceptor cell loss [59]. The connection to diabetes was first demonstrated in a study published at 2012, revealing that the damage caused to retinal ganglion cells in mice lacking Sig1R was accelerated by STZ-induced diabetes [60].

It is known that hyperglycemia and HbA1c levels over 7% are major factors predisposing patients with diabetes to vascular complications [61]. Furthermore, recent data indicates that increased glucose variability might contribute to progression of diabetic complications even if HbA1c is in the target range. Importantly, glucose variability, as assessed by continuous glucose monitoring, has been associated with damage to the neuroretina, independently of HbA1c levels, in patients with type 1 diabetes [62, 63]. Of recent, the mechanism by which glucose variability promotes neuroretinal degeneration was shown to involve activation of Müller cells. In a study of rat retinal Müller cells, the impact of glucose variability was analyzed. Cell activation was shown to differ according to basal glucose conditions, as well as subsequent exposure (constant high glucose versus alternating low/high glucose) [64].

## 4. Recent Developments in DRN Imaging

Retinal neuronal apoptosis occurs early in the disease course, causing a reduction in the thickness of inner retinal layers and of the retinal nerve fiber layer (RNFL), as may be depicted on optical coherence tomography (OCT) [6, 65–67]. In the Maastricht Study, reduced thickness of the pericentral macula was found in as early as prediabetes conditions, when compared to that of people with normal glucose metabolism, and a significant linear trend was established correlating macular thinning with severity of glucose metabolism status [68]. The conclusion that retinal neurodegenerative processes commence prior to initiation of diabetes was later supported by a study demonstrating thinner inner retinal layers and photoreceptor layers in patients with metabolic syndrome [69]. This may suggest that factors such as insulin resistance and adipose tissue-derived inflammation could cause neurodegenerative effects, independently from hyperglycemia.

Diabetes duration was found to be negatively related to the RNFL thickness in type 2 diabetic patients with early stage DR, as were BMI, triglycerides, HDL, HbA1c, and albumin-creatinine ratio [70]. Longitudinal studies of type 1 and type 2 diabetes patients with no DR or mild NPDR revealed progressive thinning of inner retinal layers [71–74]. A study using Cirrus-HD OCT for grading of en face slab OCT images of the innermost retina showed progression of damage over time and with advancing stage of DR [73]. Kim et al. also found baseline macular ganglion cell–inner plexiform layer (mGCIPL) thickness and mGCIPL thinning rate to be independent risk factors of DR progression [71].

The retinal structural alterations described have clinical implications in terms of functional deficiencies such as decreased hue discrimination, contrast sensitivity, delayed dark adaptation, and abnormal visual fields [75, 76]. Such correspondence to visual impairment was recently exemplified using an Optos OCT/SLO/microperimeter, which displayed a correlation between reduced inner and total macular thickness, and reduced microperimetric sensitivity [77].

OCT angiography (OCTA) has been extensively studied in DR, but until recently, no attempts have been made to use this technology to help elucidate the crosslink between neurodegeneration and vascular changes. Hafner et al. found a significant association between the vessel density in the deep capillary plexus of the parafovea on OCTA and the inner retinal layer thickness, mainly ganglion cell layer (GCL) and RNFL [78]. These results indicate that retinal neurodegenerative features are associated with retinal microvascular perfusion. A controlled study of eyes with no DR or mild NPDR discovered a strong positive correlation between loss of mGCIPL and reduction in vessel density from baseline to 24 months. Multivariable regression analysis showed that thinner baseline mGCIPL and greater reduction in mGCIPL thickness were significantly associated with change of vessel density [74].

## 5. Neuroprotective Therapeutic Avenues

Currently, managing DRD involves stressing the necessity of balancing blood glucose levels and targeting the microvasculopathy that is at the core of DR. Prevention and treatment of the neurodegenerative component of DRD is tragically overlooked, though the insidious loss of neurons is irreversible. The ever-growing research in the field of DRN presents opportunities to incorporate neuroprotective strategies as adjunct therapies with existing treatments for DR. Potential treatments tend to focus on one of the key players in DRN: neurotrophic factors, inflammation, and oxidative stress, though some putative therapies display mixed mechanisms. Many neuroprotective therapeutic avenues are continuously being investigated in the context of retinal disease, as has been the subject of several reviews [5, 79–84]. Our aim is to shine a light on the most recent studies of therapeutics at the forefront of the battle against DRN (Table 1).

### 5.1. Anti-inflammatory Substances

Alpha-1-antitrypsin (A1AT) commonly works as an inhibitor of serine proteases. In the context of DRD, it has been described as anti-inflammatory, involved in apoptosis avoidance and extracellular matrix remodeling and also in the protection of vessel walls and capillaries [85]. STZ-induced diabetic mice were systemically treated with A1AT (8 weekly intraperitoneal injections) and displayed a markedly reduced inflammatory status. This was evident by the downregulation of NF*κ*B, iNOS, and TNF-*α* expression, all normally increased in diabetic models and related inflammation. The treatment caused a decrease in both retinal thinning and loss of ganglion cells, thus ameliorating neurodegenerative changes [86]. In an attempt to elucidate A1AT's mechanism of action on a molecular level, it was later studied in ARPE-19 cells exposed to high glucose. A1AT normalized the levels of NF*κ*B and its targets iNOS and TNF-*α*, as well as regulated proteins related to glucose metabolism, awakened signals related to epithelial-mesenchymal transition, and normalized protein levels involved in essential RPE function [87].

Citicoline is an endogenous compound known to act as a neuroprotective agent and has been shown to be effective in the treatment of glaucoma [88]. Topical administration of citicoline in liposomal formulation in the db/db mouse model (a model for obesity-induced type 2 diabetes) prevented glial activation and neural apoptosis in the diabetic retina. In vivo, citicoline was able to ameliorate the functional abnormalities recorded on ERG in the diabetic mice. The main mechanism implicated was the inhibition of the downregulation of synaptophysin induced by diabetes and the prevention of upregulation of NF*κ*B and TNF-*α* [89].

In a retrospective study of patients with diabetic macular edema treated with intravitreal fluocinolone acetonide, neuroretinal analysis of OCT was obtained at 3-month intervals before and after treatment. In the region located 1.5 mm to 3.0 mm from the fovea, there was a statistically significant decrease in the posttreatment rate of DRN (defined as change over time of the inner neuroretinal thickness), compared with the pretreatment rate [90]. Prospective, controlled trials are necessary to further validate this effect.

### 5.2. Antioxidants

A PPAR*α* agonist used to treat dyslipidemia, fenofibrate, was found by major studies to have unprecedented therapeutic effects in DR [91–93], though the mechanism for this has not been previously elucidated (and could conceivably be caused by its lipid-normalizing effect). A later study of an experimental mouse model of type 2 diabetes indicated that neuroprotection is one of the underlying mechanisms by which fenofibrate exerts its beneficial actions in DRD [94]. Recently, in a rat model of type 1 diabetes, activation of PPAR*α* decreased retinal cell death and had a robust protective effect on retinal function. The study revealed a neuroprotective effect of PPAR*α* through improved mitochondrial function and subsequent alleviation of energetic deficits, oxidative stress, and mitochondrially mediated apoptosis [95]. As such, PPAR*α* is a promising drug target, and since then, new classes of PPAR*α* agonists were studied for proof-of-concept in vivo efficacy and preliminary pharmacokinetic assessment [96].

As previously mentioned, hyperphosphorylated tau was found to participate in DRN in a study of diabetic mice. This hyperphosphorylation, known to be induced by oxidative stress, was shown to result from an activation of glycogen synthase kinase 3*β* (GSK3*β*). Therapeutically, intravitreal administration of an short interfering RNA (siRNA) targeting tau or a specific inhibitor of GSK3*β* attenuated tau hyperphosphorylation and caused a reversion of RGC-synapse loss and restoration of visual function [43]. In a separate study, topical ocular application of ginsenoside Rg1 was shown to alleviate tau hyperphosphorylation and consequent synaptic neurodegeneration of RGCs in diabetic mice [97]. Notoginsenoside R1 was also found to have numerous mitigating effects in DRD, as oral treatment to diabetic mice caused dramatic alleviation of retinal vascular degeneration, of reduced retinal thickness, and of impaired retinal function [98]. Ginsenoside Rg1 and notoginsenoside R1 are two of the saponins extracted from the traditional Chinese medical herb *Panax notoginseng*. They are known to possess antioxidant and anti-inflammatory qualities, with resulting antidiabetic effects, also studied in the context of diabetic retinopathy [99, 100].

Extensive literature is available regarding Spermine oxidase (SMOX), a mediator of polyamine oxidation, and its role in neurodegenerative diseases in general and in neuroretinal damage specifically. SMOX inhibitors have been found to limit oxidative stress and reduce retinal neurodegeneration from various etiologies [101]. Recently, STZ-induced diabetic mice were systemically treated with MDL 72527-a SMOX inhibitor. Compared with placebo-treated diabetic mice, the treated mice displayed significantly improved ERG responses, inhibition of retinal thinning, and attenuation on RGC damage and of neurodegeneration [102].

Similarly, several other substances have been investigated for their antioxidative effect in DRN. Diabetic mice treated intravitreally with caffeic acid alkyl amide derivatives (CAF6 or CAF12), intraperitoneally with the amino acid taurine, or orally with the leucine analogue gabapentin, the acrolein-scavenging drug, 2-HDP, or the pigment astaxanthin, all exhibited reduction of oxidative stress and of neurodegenerative damage [103–107].

### 5.3. Neurotrophins and Other Neuroprotective Factors

Somatostatin (SST) is an endogenous neuroprotective peptide that is downregulated in the diabetic retina. SST downregulation is related to glial activation and neuron apoptosis, the two hallmarks of retinal neurodegeneration [108]. SST was one of the first reported topical experimental drugs to exert a neuroprotective effect [108]. In a randomized, placebo-controlled, phases II–III trial by the EUROCONDOR consortium, topical administration of SST and brimonidine was useful in arresting the progression of neurodegeneration in early DR with preexisting retinal neuro-dysfunction [109]. Amato et al. used the SST analog octreotide bound to magnetic nanoparticles and revealed it may be used as an octreotide intraocular delivery system, ensuring localization to the retina and enhanced bioactivity [110].

Ciliary neurotrophic factor (CNTF) is a member of the IL6 family of cytokines, and it supports the differentiation and survival of neurons. CNTF is also known to enhance survival of retinal photoreceptors and RGCs. CNTF delivered by encapsulated cell intraocular implants is approved for treatment of retinitis pigmentosa and of geographic atrophy [111]. In a recent study of STZ-induced diabetic rats, intravitreal injections of CNTF rescued RGCs and dopaminergic amacrine cells from neurodegeneration [48].

The accumulation of evidence regarding Sig1R's role in retinal neurodegeneration led to studies exploring its potential as a novel treatment target. Ligands for Sig1R, such as (+)-pentazocine [(+)-PTZ], were found confer marked retinal neuroprotection in vivo and in vitro [59]. In murine models of diabetic retinopathy, administration of intraperitoneal injections of (+)-PTZ resulted in significant neuroprotection, reduced evidence of oxidative stress, and preserved retinal architecture [112, 113].

The synthetic microneurotrophin BNN27 is a BBB- and BRB-permeable dehydroepiandrosterone (DHEA) derivative. It was injected intraperitoneally to STZ-induced diabetic rats and reversed the diabetes-induced glial activation and reduction of amacrine cells in a dose-dependent manner. Treatment was also found to target the inflammatory component of the disease, as it reduced proinflammatory and increased anti-inflammatory cytokine levels [114]. The neuroprotective effect to the diabetic retina was maintained with topical administration of BNN27 [115].

It has long been known that levels of brain-derived neurotrophic factor (BDNF) are reduced in DRN and that intraocular administration of BDNF counteracts diabetes-related neurodegenerative processes [116]. Of late, oral intake of eicosapentaenoic acid ethyl ester (EPA-E) was shown to ameliorate BDNF reduction and improve functional results on ERG in DRD. An EPA metabolite, 18-HEPE, induced BDNF upregulation in Müller glia cells and recovery of ERG results [117]. In a separate study, bone marrow CD133+ stem cells were intravitreally transplanted into STZ-induced diabetic mice and caused retinal BDNF levels to increase, with consequent retinal cell survival [118].

### 5.4. Mixed or Unknown Mechanisms

#### 5.4.1. Nutritional Supplements and Nutraceuticals

A variety of nutraceuticals have been studied, both in vitro and in vivo, and found to have a significant antioxidant and anti-inflammatory effect, at times reducing both the neural and vascular damage typical of DR.

Flavonoids are bioactive compounds found largely in dietary plants, aiding in the plants' protection from ultraviolet radiation, oxidants, and pathogens [119]. A high-flavonoid diet was found to be associated with lower levels of diabetic markers and reduced the prevalence of DR by 30% [120], and green tea was found to be neuroprotective in DR [121]. Over the years, a number of experimental studies showed that dietary flavonoids, such as quercetin [122], rutin [123], naringenin [124], and others, cause a reduction in oxidative stress and ameliorate inflammation and apoptosis in DRN, as was recently extensively reviewed [125, 126].

Nonflavonoid polyphenols, such as curcumin and resveratrol, have been shown to exert antiapoptotic effects on the retina of diabetic rat models, with attenuation of retinal thinning, among other neuroprotective influences. Both these substances have been reported to inhibit apoptosis by stimulating autophagy [127–130].

A variety of studies have examined the possible protective role of Müller cell-autophagy in DR [131, 132]. Inhibition of autophagy increased retinal cell apoptosis induced by high glucose [133], whereas its activation could protect Müller cells from high glucose-induced apoptosis [134]. Epigallocatechin-3-gallate (EGCG) is a major polyphenol in green tea that has attracted attention as a potential therapy for various pathologies, including apoptosis of retinal neurons [135, 136]. In a recent study, retinal Müller cells in high glucose conditions treated with EGCG showed activation of autophagy machinery and reestablishment of cargo degradation, which protected the cells from apoptosis. EGCG could increase the ability of cells to proliferate by increasing autophagy. In a mouse model of diabetic retinopathy, EGCG treatment reduced the reactive gliosis of Müller cells and decreased retinal damage [137].

As mentioned previously, the antioxidative effect of notoginsenoside R1 [98] and the neurotrophic effect of EPA-A [117, 118] are also the subject of recent research, as are other nutraceuticals and nutritional supplements [138–140].

#### 5.4.2. Therapeutic Targets of the Renin-Angiotensin System (RAS) in DRN

The role of RAS in the development and progression of DRD is well established [141–144]. In recent years, research shed more light in regard to the relationship between RAS and the neurovascular unit [145]. Some researchers explored the therapeutic feasibility of RAS-related substances for DRN prevention or control. Retinal explants treated with angiotensin II demonstrated a 40% reduction in RGC survival, compared with vehicle [146]. Treatment of STZ-induced diabetic rats with telmisartan, an angiotensin II type 1 receptor blocker, caused elevated levels of neurotrophic factors in the sera and retinas compared with untreated rats, as well as an increase of endogenous antioxidant glutathione content and decreased signs of apoptosis in diabetic retina [147]. Treatment of STZ-induced diabetic rats with an angiotensin-converting enzyme 2 (ACE2) activator significantly reduced the apoptotic cell death of RGCs compared with untreated diabetic rats [148]. Verma et al. used engineered probiotic species as live vector for oral delivery of human ACE2 with enhanced tissue bioavailability, blocking RGC loss in two mouse models of diabetic retinopathy, while also reducing retinal inflammatory cytokine expression and the number of acellular capillaries [149].

#### 5.4.3. Additional Novel Therapeutic Pathways

One of the more promising therapeutic agents investigated is glucagon-like peptide-1 (GLP-1) and GLP-1 receptor agonist (GLP-1RA) liraglutide. Liraglutide is currently used to treat type 2 diabetes and is known to have neuroprotective effects. It was previously reported that topical administration of GLP-1 or GLP-1RAs prevented DRN and early vascular leakage in early treatment of diabetic mice (treated at the age of 10 weeks, before retinal abnormalities are detected) [150, 151]. The same group went on to prove that the treatment with topical GLP-1 at a later stage (24 weeks) could revert the retinal neurodegeneration induced by long-term diabetes. The treatment generated anti-inflammatory effects, anti-apoptotic effects, anti-VEGF, and even neuroregenerative ones [152]. Liraglutide also incurs antiendoplasmic reticulum stress and-oxidative stress effects, in its protective action against DRN [153], as well as reversal of hyperphosphorylated tau-triggered RGCs synaptic degeneration [154].

Another drug to show encouraging results is lamivudine (3TC), a newly discovered Purinergic Receptor P2X 7 (P2rx7) inhibitor. P2rx7 is upregulated in diabetes and its inhibition via oral treatment of lamivudine reversed retinal neuronal, as well as vascular damage, incurred by diabetes. This was evident as neuroglial function on ERG was maintained, the number of GABAergic amacrine cells was improved, and the formation of acellular capillaries in the retina was prevented [155].

It has been suggested that endothelin 1 (ET-1) is involved in the development of diabetic retinal microvasculopathy [156]. Endothelin B-receptors activation mediates retinal neurodegeneration, but this was not previously proved to occur in diabetes. Recently, it was found that upregulation of ET-1 and its receptors is an early event in the diabetic retina. Topical administration of bosentan (a dual endothelin receptor antagonist) in diabetic (db/db) mice was shown to result in a significant decrease of reactive gliosis and apoptosis. Anti-inflammatory, as well as anti-VEGF mechanisms, was implicated in bosentan's auspicious effects [157].

Lastly, as allegations of an association between diabetes and glaucoma, on fluctuations in intraocular pressure (IOP), continue to arise [158], the role of serial IOP changes in DRN was investigated in STZ-induced diabetic rats. Diabetic rats exhibited higher fluctuations of IOP than normal controls or diabetic rats treated with brinzolamide and latanoprost ophthalmic solutions. IOP-lowering drugs reduced RGC-apoptosis and were suggested to decrease intermittent mechanical stress, glial activation, axoplasmic flow, and eventually neurodegeneration [159].

## 6. Discussion

Recent progress has provided increasing evidence of the importance of DRN in progression of DR and DRD. Therefore, improved understanding of its mechanisms, as well as novel approaches for diagnosis and treatment, is needed.

**Figure 1:**
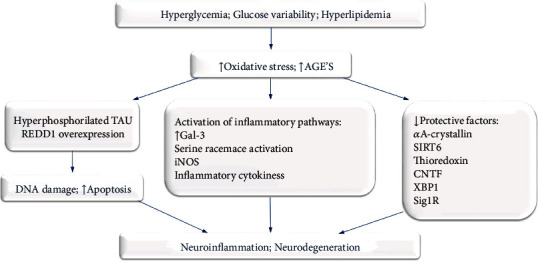
Key mechanisms and recent discoveries in diabetic retinal neurodegeneration.

Several new molecular biomarkers of DRN have been identified recently in experimental models of diabetes. These are proteins involved in inflammation, oxidative stress, apoptosis, cell survival, endoplasmic reticulum stress response, aging, and other cell processes (Figure 1). These novel biomarkers of DRN include galectin-3 [18, 34–36] serine racemase (SRR) and its product, D-serine [37–40], REDD1 [41, 42], hyperphosphorylated tau [43], *α*A-crystallin [44], SIRT6 [45], thioredoxin [46, 47], CNT [48], and XBP1 [49]. They might become promising targets for timely diagnosis and treatment of DRN in the future.

Among potentially modifiable clinical factors associated with DRN, glucose variability has emerged as an important contributor to DRD, even in the presence of HbA1c levels corresponding to “well controlled diabetes” [62–64].

Diagnostic approaches of DRN include several modalities. Application of OCT has been shown to be effective in demonstrating structural changes in DRN, such as thinning of the retinal layers [6, 65–74]. For demonstration of functional deficiencies characteristic of DRN, an Optos OCT/SLO/microperimeter has been applied [77]. Finally, OCTA has been used in studies to evaluate a crosslink between neurodegeneration and vascular changes in retina [74, 78].

Several novel therapeutic approaches of DRN have been applied recently. Promising results have been obtained in experimental models of DRN with anti-inflammatory agents, such as alpha-1-antitrypsin (A1AT) [85–87], citicoline [88, 89], epigallocatechin-3-gallate (EGCG) [135–137]. Intravitreal fluocinolone acetonide, used to treat patients with diabetic macular edema, achieved a positive effect on the inhibition of DRN [90]. Of the antioxidants, fenofibrate and PPAR*α* activation demonstrated neuroprotective effects on DRD, in both clinical and experimental studies, and results of studies with novel PPAR*α* agonists in retinal diseases are forthcoming [91–96]. Several novel compounds targeting protein tau hyperphosphorylation are under investigation in experimental studies [43, 97, 98]. Other compounds with antioxidative actions under investigation in DRN include SMOX inhibitors [101, 102], caffeic acid alkyl amide derivatives, taurine, gabapentin, and others [103–107].

Of the neurotrophic and neuroprotective substances, CNTF [48, 111], Sig1R [54, 59, 112, 113], and synthetic microneurotrophin BNN27 [114, 115], were shown to have neuroprotective properties and ability to reduce neurodegeneration in diabetic animals. Several studies have targeted BDNF reduction via intraocular BDNF administration [116], oral intake of eicosapentaenoic acid ethyl ester [116], and intravitreal bone marrow CD133+ stem cells transplantation, leading to improved retinal cell survival [118]. Topical administration of SST was tested in a randomized, placebo-controlled, phases II–III trial and could inhibit the neurodegenerative process in early DR with preexisting retinal neuro-dysfunction [109].

Among substances with unknown or mixed mechanisms, flavonoids and other nutritional supplements show great promise [125–129, 137–140]. Substances that alter the RAS have been extensively studied, with exciting new prospects [147–149]. Other promising agents with neuroprotective effects in animal models of DRN include GLP-1RA liraglutide [150–154], lamivudine (P2rx7 inhibitor) [155], endothelin-1 receptor antagonists [156, 157], and agents lowering intraocular pressure [158, 159].

To conclude, an impressive body of evidence is accumulating regarding DRN's role in the ocular damage caused by diabetes. Disruptions of glucose and lipid status generate oxidative stress and increase formation of advanced glycation end products, setting in motion several pathologic processes, including amplification of inflammatory pathways, impediment of neuroprotective pathways, and induction of DNA damage and apoptosis. As information regarding these cellular and molecular mechanisms is revealed and as diagnostic modalities evolve, expeditious detection of DRN is made feasible. Promising results have been attained with various substances, including antioxidants, neuroprotective factors, and anti-inflammatory substances, in an attempt to attenuate DRN. Further studies are needed to facilitate clinical implementation of novel options for timely diagnosis, prevention, and treatment of this pivotal component of DRD.

The long-awaited implementation of this therapeutic approach cannot materialize without robust animal models of DR, accurately mimicking the human disease. Translating experimental success from animals to humans is often hurdled by conceptual and methodological challenges, such as the timing of the therapeutic intervention, participant follow-up, disease heterogeneity, effective drug delivery, and selecting reproducible and clinically important trial endpoints. In addition, major efforts should be devoted to standardizing methods for screening and monitoring of neurodegeneration, to ensure uniformity across studies. It is essential that a set of guidelines is established for such experimental and clinical studies. Lastly, combination therapies of DRN merit further research, as many of these approaches have potentially complimentary mechanisms, which may produce synergistic effects, thereby improving the overall neuroprotective effect.

While there is still a way to go, taking into account the volume of information accumulated thus far, the question is no longer “Will treatment of DRD include DRN-targeted therapies?”, the question now is “How and when?”. A multidisciplinary collaborative effort is required in order to address this issue and offer hope that functional vision may be sustained throughout the lives of the diabetic patients.

## Figures and Tables

**Table 1:** Novel therapeutic approaches to diabetic retinal neurodegeneration in experimental studies.

Anti-inflammatory substances	Antioxidants	Neurotrophins and other neuroprotective factors	Mixed or unknown mechanisms
Alpha-1-antitrypsin	Fenofibrate and other PPAR*α* agonists	Somatostatin	Flavonoids and other nutraceuticals
Citicoline	Inhibitors of protein tau hyperphosphorylation: ginsenoside Rg1 Notoginsenoside R1 siRNA	Ciliary neurotrophic factor	Angiotensin II type 1 receptor blockers
Fluocinolone acetonide	Spermine oxidase inhibitors	Sigma1 receptor	Angiotensin-converting enzyme 2 activators
	Caffeic acid alkyl amide derivatives	Synthetic microneurotrophin BNN27	GLP-1 receptor agonists
	Taurine	Brain-derived neurotrophic factor	Lamivudine
	Gabapentin	Eicosapentaenoic acid ethyl ester	Endothelin-1 receptor antagonists
	Acrolein-scavenging 2-HDP	Intravitreal bone marrow CD133+ stem cells transplantation	Intraocular pressure-lowering agents
	Astaxanthin		
